# Psychological predictors of sport dropout in adolescents: a latent profile analysis of risk profiles and R3STEP auxiliary regression

**DOI:** 10.3389/fpsyg.2026.1972994

**Published:** 2026-10-02

**Authors:** Tianzhi Zhan, Lu Liu

**Affiliations:** College of Physical Education and Health, Jiangsu Normal University, Xuzhou, China

**Keywords:** adolescent athletes, amotivation, athlete burnout, competitive trait anxiety, latent profile analysis, person-centered approach, R3STEP, sport dropout

## Abstract

Understanding why adolescents discontinue organized sport is central to sustaining the physical and mental health benefits of sport participation. Most research has examined psychological correlates of dropout one variable at a time, yet burnout, amotivation, and competitive anxiety may co-occur in meaningful configurations. This study applied a person-centered latent profile analysis (LPA) to identify psychological risk profiles among adolescent athletes and used the R3STEP three-step approach to examine whether dropout status was associated with baseline profile membership over a 12-month follow-up. A total of 864 competitive adolescent athletes (46.5% girls; mean age = 14.6 years, SD = 1.8) from Jiangsu Province, China, completed measures of athlete burnout (emotional/physical exhaustion, reduced sense of accomplishment, sport devaluation), amotivation, and multidimensional competitive anxiety (somatic, worry, concentration disruption). Dropout status was recorded 12 months later. Model selection indices supported a three-profile solution: a low-risk profile (52.8%), characterized by uniformly favorable scores; a moderate-risk profile (33.4%); and a high-risk profile (13.8%), marked by pronounced elevations across all seven indicators. Twelve-month dropout rates rose monotonically across profiles (8.6%, 19.4%, and 35.3%, respectively), χ^2^(2) = 45.87, *p* < 0.001. In R3STEP analyses controlling for age, sex, and weekly training hours, dropouts were 3.86 times more likely to belong to the high-risk rather than the low-risk profile (OR = 3.86, *p* < 0.001) and 1.94 times more likely to belong to the moderate-risk profile (OR = 1.94, *p* < 0.01). Older age and greater weekly training hours were also associated with higher-risk membership. These findings indicate that burnout, amotivation, and anxiety cluster within identifiable subgroups of adolescent athletes, and that membership in a high-risk psychological profile is strongly associated with subsequent sport dropout. These profiles may inform the future development of risk-assessment approaches for retaining adolescent athletes, pending prospective validation of their predictive performance.

## 1 Introduction

Organized youth sport confers physical, psychological, and social benefits, yet a substantial proportion of adolescents withdraw from competitive sport each year (Crane and Temple, 2015; Balish et al., 2014). Systematic reviews estimate that roughly one third of youth sport participants discontinue participation annually, with attrition intensifying during adolescence, and dropout is associated with reduced physical activity, diminished wellbeing, and loss of developmental opportunities (Fraser-Thomas et al., 2008; Crane and Temple, 2015). Identifying the psychological configurations that precede withdrawal is therefore a priority for sport organizations committed to athlete retention and long-term participation (Côté, 1999; Scanlan et al., 1993).

Three psychological constructs have emerged as consistently relevant to dropout. First, athlete burnout—a syndrome comprising emotional and physical exhaustion, a reduced sense of accomplishment, and devaluation of sport (Raedeke, 1997; Raedeke and Smith, 2001)—has been linked to withdrawal intentions and attrition in competitive samples (Gould et al., 1996a,b; Gustafsson et al., 2007; Goodger et al., 2007; Madigan et al., 2015). Burnout is theorized to arise from chronic stress entrapment, in which athletes remain bound to sport despite eroding rewards (Raedeke, 1997; Smith, 1986; Coakley, 1992). Second, amotivation—the relative absence of intentional behavior regulation in self-determination theory (Deci and Ryan, 2000; Ryan and Deci, 2017)—reflects needs thwarting in the sporting environment (Bartholomew et al., 2011) and is among the strongest motivational predictors of dropout: prospective studies show that athletes with low self-determined motivation are disproportionately likely to withdraw within subsequent seasons (Sarrazin et al., 2002; Guillet et al., 2002; Pelletier et al., 2001). Third, competitive trait anxiety, including somatic symptoms, cognitive worry, and concentration disruption (Smith et al., 2006), represents an aversive competitive experience that erodes enjoyment and fuels withdrawal ideation (Gould et al., 1996a,b; Raedeke and Smith, 2004).

Although informative, this literature is dominated by variable-centered analyses that estimate average associations between single constructs and dropout. Variable-centered approaches assume that the sample is homogeneous with respect to the covariation structure and can therefore obscure subgroups of athletes whose configurations of risk differ qualitatively (Marsh et al., 2009; Collins and Lanza, 2010). A complementary person-centered perspective treats configurations of attributes as holistic profiles and asks how many such profiles exist, who belongs to them, and how they relate to outcomes. Person-centered studies in sport and physical education settings have shown that motivational and burnout profiles display theoretically coherent shapes and that profile membership is associated with outcomes beyond the contributions of individual variables (Wang et al., 2002; Lonsdale et al., 2009; Hodge et al., 2008; DeFreese and Smith, 2013). However, few studies have combined burnout dimensions, amotivation, and competitive anxiety simultaneously within a single profile structure, and still fewer have linked these profiles to objectively recorded dropout rather than withdrawal intentions (Balish et al., 2014).

These issues are especially salient in the Chinese competitive sport system, where large numbers of adolescents enter sports schools and club training programs that combine academic schooling with intensive, early-specialized preparation for regional and provincial competition. Such environments offer high-quality coaching and clear progression pathways but also concentrate training loads, selection pressure, and performance evaluation during a developmental period in which athletic identity and motivation for sport are actively constructed. Psychological research on Chinese adolescent athletes has grown rapidly, yet person-centered evidence linking burnout, amotivation, and anxiety configurations to actual withdrawal remains scarce, and dropout findings obtained in Western settings may not transfer directly to the Chinese system (Balish et al., 2014; Crane and Temple, 2015). Establishing whether multidimensional risk profiles are prospectively associated with dropout in this population is therefore both theoretically and practically consequential.

Latent profile analysis (LPA) is well-suited to this task. LPA identifies unobserved subgroups (latent profiles) characterized by distinct patterns of means on continuous indicators, with class enumeration guided by information criteria, entropy, and likelihood ratio tests (Nylund et al., 2007; Jung and Wickrama, 2008; Tein et al., 2013). A methodological challenge in LPA is that the classification of individuals into profiles is uncertain, and naïvely relating observed class membership to outcomes attenuates associations. The R3STEP auxiliary procedure (Asparouhov and Muthén, 2014) addresses this problem by estimating the relationship between latent class membership and auxiliary variables while correcting for classification error, and it therefore provides an appropriate framework for examining whether psychologically defined profiles prospectively relate to dropout.

The present study pursued three research questions in a sample of 864 competitive adolescent athletes from Jiangsu Province, China, followed for 12 months. First, how many psychological risk profiles can be identified from seven indicators spanning the three burnout dimensions, amotivation, and the three competitive anxiety dimensions, and what shapes do these profiles take (RQ1)? Second, do the identified profiles differ in their rates of sport dropout over the follow-up period (RQ2)? Third, does dropout status relate to profile membership after accounting for age, sex, and weekly training hours in an R3STEP auxiliary regression (RQ3)? Based on prior person-centered evidence and theory, we expected a small number of profiles (three or four) ordered along a global risk dimension, with a minority of athletes exhibiting a high-risk configuration (Gustafsson et al., 2007; Wang et al., 2002). Regarding profile shape, two possibilities were considered: a transdiagnostic prediction, grounded in self-determination theory and stress-based burnout models, that burnout, amotivation, and anxiety would co-occur in a convergent configuration reflecting common needs thwarting and chronic strain (Deci and Ryan, 2000; Smith, 1986), and a domain-specific prediction that the three risk domains would instead form relatively distinct specialized profiles (Marsh et al., 2009); we favored the former. Finally, we expected athletes in higher-risk profiles to show higher dropout rates (Sarrazin et al., 2002; Raedeke, 1997). Because the R3STEP procedure regresses profile membership on auxiliary variables, the resulting odds ratios express associations between dropout status and baseline profile membership; accordingly, the findings are interpreted in associational terms throughout this article.

## 2 Materials and methods

### 2.1 Participants

Participants were 864 competitive adolescent athletes (402 girls, 46.5%; 462 boys, 53.5%) recruited from 21 sports schools and competitive clubs in Xuzhou and neighboring cities of Jiangsu Province, China. Of 936 athletes invited to participate, 903 returned questionnaires, and 39 were excluded for incomplete responses on the analysis variables, yielding the final sample of 864 complete cases; excluded athletes did not differ meaningfully from included athletes in age or sex distribution. Their ages ranged from 12 to 17 years (mean age = 14.6 years, SD = 1.8). Athletes represented eight sports, including athletics, basketball, soccer, volleyball, table tennis, badminton, swimming, and tennis, and all trained systematically for regional or provincial competition, with weekly training hours averaging 12.4 (SD = 4.6). Sample size considerably exceeds the minimum *N* required for adequate power to detect small classes in LPA with seven continuous indicators (Tein et al., 2013).

### 2.2 Measures

#### 2.2.1 Athlete burnout

Burnout was measured with the 15-item Athlete Burnout Questionnaire (ABQ; Raedeke and Smith, 2001), comprising three five-item subscales: emotional and physical exhaustion (e.g., “I am exhausted by the mental and physical demands of my sport”), reduced sense of accomplishment (e.g., “I am not achieving much in my sport and I wonder if I should just give up”), and sport devaluation (e.g., “I have a negative feeling toward my sport”). Items were rated from 1 (almost never) to 5 (almost always). Cronbach’s alphas were 0.89, 0.85, and 0.87 for the three subscales, respectively. Athletes completed the Chinese-language version of the instrument, whose psychometric properties have been supported in Chinese athlete samples (Liu et al., 2022).

#### 2.2.2 Amotivation

Amotivation was assessed with the four-item amotivation subscale of the revised Sport Motivation Scale (SMS-II; Pelletier et al., 2013), which revises the original Sport Motivation Scale (Pelletier et al., 1995) and is grounded in self-determination theory (Deci and Ryan, 2000). A sample item is “I participate in my sport but I wonder why I continue.” Respondents used the response scale recommended for the instrument, and subscale scores were computed as item means. Cronbach’s alpha was 0.82. The Chinese-language version of the SMS-II has demonstrated satisfactory reliability and validity among Chinese athletes (Li et al., 2018).

#### 2.2.3 Competitive trait anxiety

Competitive trait anxiety was measured with the 21-item Sport Anxiety Scale-2 (SAS-2; Smith et al., 2006), which assesses somatic anxiety (e.g., “My body feels tense”), worry (e.g., “I worry I will not play well”), and concentration disruption (e.g., “It is hard for me to focus on what I am supposed to do”), each with seven items. Cronbach’s alphas were 0.88, 0.87, and 0.86 for the three subscales, respectively. Athletes completed the Chinese-language SAS-2, which has shown robust psychometric properties in Chinese adolescent and student athletes (Li et al., 2023; Zhang et al., 2023).

All three instruments are established measures with validated factor structures in athlete samples; subscale scores were therefore used directly as observed indicators in the latent profile analysis, and no study-specific confirmatory factor analysis was conducted.

#### 2.2.4 Sport dropout

Dropout was recorded 12 months after the baseline assessment by consulting registration and training enrollment records provided by the participating schools and clubs. An athlete was classified as having dropped out if he or she had discontinued organized training and competition in his or her primary sport and had not re-enrolled in any other organized sport at follow-up; re-enrollment in another organized program was identified through the same institutional enrollment records, which cover registrations within the regional sport system. Following this criterion, 137 athletes (15.9%) had dropped out, and 727 (84.1%) remained active.

### 2.3 Procedure

Baseline data were collected during the autumn semester of 2024 in quiet team meeting rooms, with trained research assistants administering the questionnaires according to a standardized protocol. Athletes completed the ABQ, the SMS-II amotivation subscale, and the SAS-2, along with a demographic form covering age, sex, sport, competitive level, and weekly training hours (self-reported). Follow-up dropout status was ascertained 12 months later from institutional records, without further athlete contact. The entire procedure was carried out in accordance with the ethical principles of the Declaration of Helsinki and was approved by the Ethics Committee of Jiangsu Normal University. Participation was voluntary; written informed consent was obtained from a parent or legal guardian of every athlete together with the athlete’s own assent, and data were anonymized prior to analysis.

### 2.4 Data analysis

Questionnaires containing incomplete responses on the analysis variables were excluded prior to analysis, and all reported analyses are based on complete cases. Analyses were conducted in Mplus 8.3 (Muthén and Muthén, 1998–2017). Latent profile analysis was estimated with maximum likelihood using one- through five-class solutions. Model selection followed recommended practice (Nylund et al., 2007; Jung and Wickrama, 2008): lower values on the Akaike information criterion (AIC), Bayesian information criterion (BIC), and sample-size adjusted BIC (SSA-BIC), higher entropy, and significant Lo–Mendell–Rubin adjusted likelihood ratio tests (LMR-LRT) and bootstrapped likelihood ratio tests (BLRT) favored additional classes, with interpretability, class size (minimum 5% of the sample), and theoretical coherence serving as final criteria. All seven indicators were standardized to facilitate comparison of profile shapes. Following model selection, the R3STEP procedure (Asparouhov and Muthén, 2014) was used to regress latent profile membership on dropout status (1 = dropped out, 0 = persisted), age, sex, and weekly training hours. R3STEP corrects for the uncertainty of latent class assignment when relating classes to auxiliary variables, and odds ratios from these multinomial regressions index the increase in odds of membership in a given profile relative to the low-risk profile that is associated with the auxiliary predictor, net of the other predictors in the model. Profile differences in raw dropout rates were additionally examined with a chi-square test of independence. Age, sex, and weekly training hours were selected a priori as covariates because they are established demographic and training-load correlates of dropout; sport type and competitive level were not included, because with eight sports and multiple competitive levels distributed across three profiles the resulting cells would be too sparse for stable estimation. Models were estimated with 1,000 random starts and 250 final-stage optimizations, and the best log-likelihood value was replicated across starts, indicating that the retained solutions were not local maxima. Because the R3STEP parameterization regresses profile membership on the auxiliary variables, the resulting odds ratios express how strongly dropout status is associated with prior profile membership rather than the probability of dropout within each profile; they are therefore interpreted throughout as prospective associations.

## 3 Results

### 3.1 Latent profile enumeration

Fit indices for the one- through five-class solutions are reported in Table 1. Information criteria decreased across the one- through five-class solutions; the three-class solution was selected because entropy peaked at three classes (0.91), the LMR-LRT was non-significant for the four-class solution, and the additional classes beyond three each contained fewer than 5% of participants and differed from existing classes in degree rather than in shape. The four-class solution added a small class that resembled the moderate-risk profile in shape, and the five-class solution was similarly unstable. Following recommended enumeration practice (Nylund et al., 2007), the three-class solution was retained.

**TABLE 1:** Fit indices for one- through five-class latent profile solutions (*N* = 864).

Classes	AIC	BIC	SSA-BIC	Entropy	LMR-LRT *p*	BLRT *p*
1	24587.32	24659.85	24617.44	–	–	–
2	23688.41	23784.65	23727.61	0.86	<0.001	<0.001
3	23241.58	23361.53	23289.86	0.91	0.003	<0.001
4	23198.74	23342.40	23256.10	0.89	0.187	0.021
5	23167.29	23334.66	23233.72	0.87	0.264	0.058

AIC, Akaike information criterion; BIC, Bayesian information criterion; SSA-BIC, sample-size adjusted BIC; LMR-LRT, Lo–Mendell–Rubin adjusted likelihood ratio test; BLRT, bootstrapped likelihood ratio test. The three-class solution was retained based on the joint consideration of fit indices, entropy, statistical tests, class size, and interpretability.

### 3.2 Description of the three profiles

Standardized indicator means for the three profiles are presented in Table 2 and visualized in Figure 1. The largest profile, labeled low-risk (52.8%, *n* = 456), was characterized by below-average scores on all seven indicators, with particularly low scores for sport devaluation and amotivation. The second profile, labeled moderate-risk (33.4%, *n* = 289), showed slightly elevated scores across all indicators, hovering between 0.1 and 0.2 SD above the sample mean. The smallest profile, labeled high-risk (13.8%, *n* = 119), displayed marked elevations on every indicator, exceeding one full standard deviation above the mean for sport devaluation (1.64), exhaustion (1.52), and amotivation (1.41). Across profiles, burnout, amotivation, and anxiety indicators rose and fell together, supporting a convergent risk configuration rather than distinct specialized profiles (RQ1).

**TABLE 2:** Profile sizes and standardized indicator means for the three-class solution.

Indicator	Low-risk (52.8%)	Moderate-risk (33.4%)	High-risk (13.8%)
Emotional/physical exhaustion	−0.62	0.18	1.52
Reduced sense of accomplishment	−0.38	0.11	1.19
Sport devaluation	−0.71	0.21	1.64
Amotivation	−0.58	0.15	1.41
Somatic anxiety	−0.55	0.09	1.31
Worry	−0.47	0.10	1.22
Concentration disruption	−0.52	0.13	1.18

Values are standardized (z-score) indicator means within each profile. Percentages in column headers represent the proportion of the sample classified in each profile.

**FIGURE 1:**
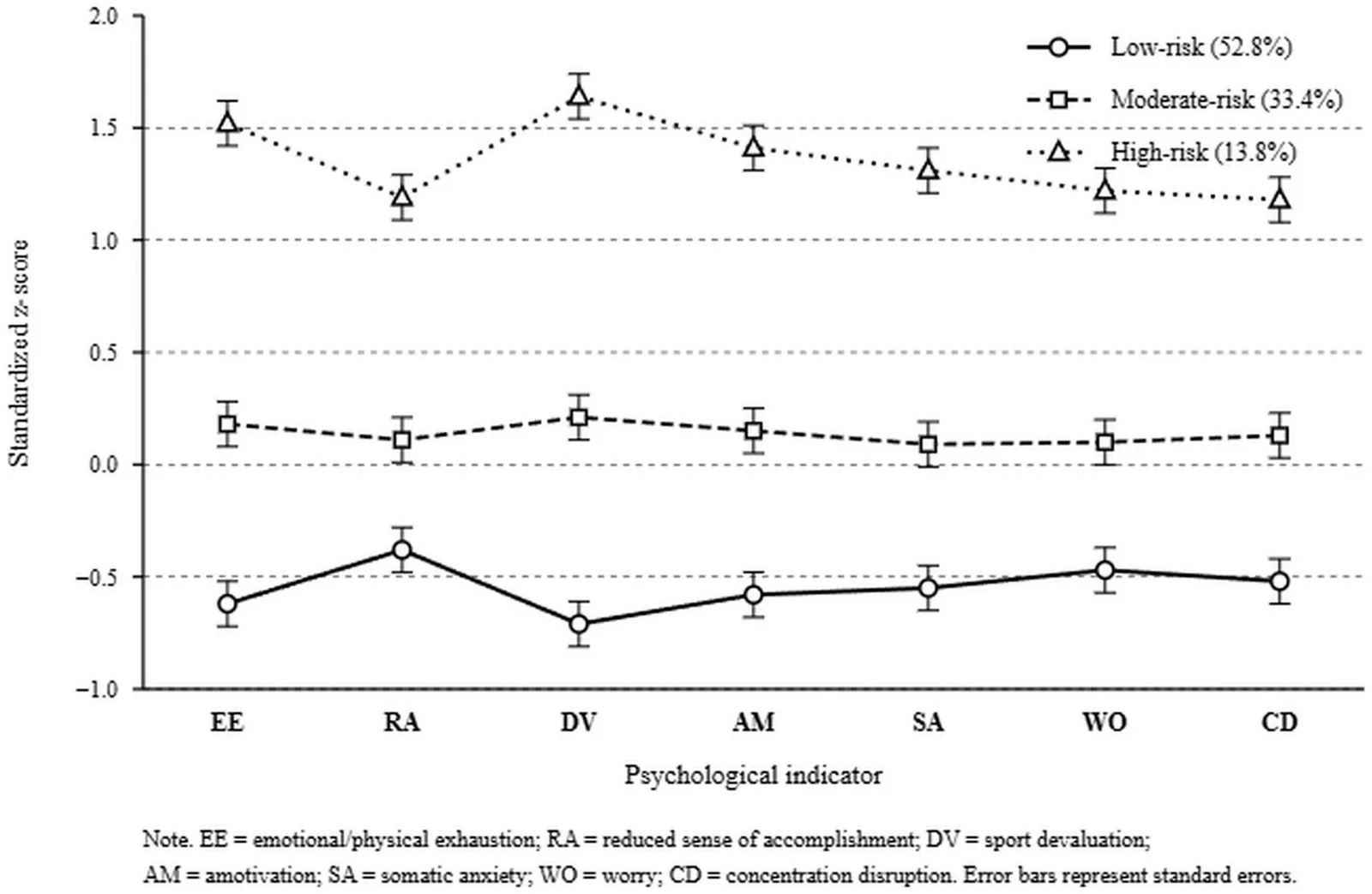
Standardized (z-score) indicator means of the seven psychological indicators for the three latent profiles: low-risk (52.8%), moderate-risk (33.4%), and high-risk (13.8%). Indicators are emotional/physical exhaustion (EE), reduced sense of accomplishment (RA), sport devaluation (DV), amotivation (AM), somatic anxiety (SA), worry (WO), and concentration disruption (CD). Error bars represent standard errors.

### 3.3 Profile differences in dropout

Twelve-month dropout rates differed substantially across profiles (Table 3). Among low-risk athletes, 39 of 456 (8.6%) had dropped out; among moderate-risk athletes, 56 of 289 (19.4%) had dropped out; and among high-risk athletes, 42 of 119 (35.3%) had dropped out. The association between profile membership and dropout was significant, χ^2^(2) = 45.87, *p* < 0.001, with a monotonic increase in dropout risk from the low-risk to the high-risk profile (RQ2).

**TABLE 3:** Twelve-month dropout rates across latent profiles.

Profile	*n*	Dropouts (*n*)	Dropout rate (%)	95% CI (%)
Low-risk	456	39	8.6	[6.3, 11.6]
Moderate-risk	289	56	19.4	[15.0, 24.5]
High-risk	119	42	35.3	[26.9, 44.6]

CIs, confidence intervals for proportions. Total dropouts, 137 of 864 athletes (15.9%). The association between profile membership and dropout was significant, χ^2^(2) = 45.87, *p* < 0.001.

### 3.4 R3STEP auxiliary regression

Results of the R3STEP multinomial logistic regression are reported in Table 4, with the low-risk profile as the reference category. Dropout status was significantly associated with membership in both risk profiles: relative to persisters, athletes who dropped out were 3.86 times more likely to belong to the high-risk profile than to the low-risk profile (OR = 3.86, 95% CI [2.31, 6.44], *p* < 0.001) and 1.94 times more likely to belong to the moderate-risk profile (OR = 1.94, 95% CI [1.22, 3.09], *p* < 0.01). Among the covariates, older age was associated with membership in the high-risk profile (OR = 1.21, 95% CI [1.02, 1.43], *p* < 0.05), and greater weekly training hours was associated with membership in both the high-risk (OR = 1.14, 95% CI [1.05, 1.24], *p* < 0.01) and moderate-risk profiles (OR = 1.09, 95% CI [1.01, 1.17], *p* < 0.05). Sex was unrelated to profile membership (RQ3). In combination, these results indicate that the psychological risk configuration captured by the profiles was consistently associated with subsequent dropout over and above age, sex, and training load.

**TABLE 4:** R3STEP multinomial logistic regression of latent profile membership on dropout and covariates (reference: low-risk profile).

Predictor	OR (high vs. low)	95% CI	*P*	OR (moderate vs. low)	95% CI	*P*
Dropout (1 = yes)	3.86	[2.31, 6.44]	<0.001	1.94	[1.22, 3.09]	<0.01
Age (years)	1.21	[1.02, 1.43]	<0.05	1.08	[0.94, 1.25]	0.274
Sex (girl = 1)	1.27	[0.79, 2.04]	0.325	1.11	[0.77, 1.59]	0.583
Weekly training hours	1.14	[1.05, 1.24]	<0.01	1.09	[1.01, 1.17]	<0.05

OR, odds ratio; CI, confidence interval. Odds ratios index the increase in odds of belonging to the given profile versus the low-risk profile associated with a one-unit increase in the predictor (dropout coded 1, dropped out; 0, persisted). Estimates correct for latent class classification error via the R3STEP procedure.

## 4 Discussion

This study applied a person-centered perspective to the problem of adolescent sport dropout. Using latent profile analysis with seven indicators spanning athlete burnout, amotivation, and multidimensional competitive anxiety, we identified three psychological risk profiles in a large sample of Chinese competitive adolescent athletes, and we linked these profiles to dropout recorded over a 12-month follow-up using an R3STEP auxiliary regression that corrects for classification error. The findings clarify how psychological risks cluster among adolescents and demonstrate that a convergent high-risk configuration is a strong prospective correlate of withdrawal. The designation of these profiles as risk profiles is justified not by their psychological composition alone but by their prospective association with dropout demonstrated in the present data.

Addressing RQ1, the three-profile solution—low-risk (52.8%), moderate-risk (33.4%), and high-risk (13.8%)—is consistent with person-centered research in both sport and educational settings, which typically recovers a majority adaptive group, one or two intermediate groups, and a small multiply-burdened group (Wang et al., 2002; Marsh et al., 2009). The convergent shape of the profiles is theoretically informative. Exhaustion, reduced accomplishment, devaluation, amotivation, somatic anxiety, worry, and concentration disruption did not combine into specialized subtypes; instead, they rose and fell in tandem. This coherence accords with self-determination theory, in which frustrated basic needs simultaneously undermine the quality of motivation and generate ill-being (Deci and Ryan, 2000; Ryan and Deci, 2017; Bartholomew et al., 2011; Lonsdale et al., 2009; Hodge et al., 2008), and with stress-based models of burnout that treat exhaustion, devaluation, and anxiety as facets of a common chronic strain process (Smith, 1986; Raedeke, 1997). The observed proportion of high-risk athletes (13.8%) is somewhat higher than prevalence estimates of severe burnout among adolescent elite athletes (Gustafsson et al., 2007), plausibly because profiles were defined by multiple risk domains rather than burnout alone.

Addressing RQ2 and RQ3, dropout rates increased monotonically across the profiles, and the R3STEP analyses confirmed that dropouts were nearly four times more likely than persisters to belong to the high-risk profile relative to the low-risk profile, net of age, sex, and training hours. This association is of a magnitude comparable to the moderate prospective links that amotivation and low self-determined motivation have shown with withdrawal in previous research (Sarrazin et al., 2002; Guillet et al., 2002; Balish et al., 2014). The result suggests that the joint configuration of burnout, amotivation, and anxiety carries information beyond any single indicator, precisely because withdrawal is typically the end point of accumulating strains across motivational and wellbeing domains (Raedeke, 1997; Crane and Temple, 2015). From a stress perspective, athletes in the high-risk profile appear to experience the entrapment pattern described by Raedeke (1997), Coakley (1992): continued investment under eroding attraction and accomplishment, with anxiety maintaining vigilance and exhaustion depleting capacity, until sport participation is finally abandoned.

Beyond the specific findings, the study makes two broader contributions. Theoretically, it demonstrates complementarity between variable- and person-centered approaches to dropout research (Marsh et al., 2009). Variable-centered studies estimate how a single construct relates to withdrawal on average; the present profiles show that burnout, amotivation, and anxiety operate as a coordinated configuration whose upper tail carries disproportionate risk. This reframing is consistent with developmental proposals that dropout is the endpoint of accumulating strains rather than the product of any single deficit (Fraser-Thomas et al., 2008; Crane and Temple, 2015). Methodologically, the study illustrates the value of the R3STEP auxiliary framework for sport research: by correcting for classification error, R3STEP yields less biased estimates of profile–outcome associations than approaches that treat observed class assignments as error-free (Asparouhov and Muthén, 2014; Tein et al., 2013), and its application here provides a template for future prospective person-centered studies of athlete wellbeing.

The covariate findings add nuance. Older adolescents were more likely to occupy the high-risk profile, consistent with evidence that dropout risk intensifies across adolescence as competing demands and identity decisions accumulate (Fraser-Thomas et al., 2008; Crane and Temple, 2015). Greater weekly training hours were also associated with higher-risk membership, echoing concerns about early specialization and heavy training loads in youth sport, although training load was measured through self-report and was not the focus of this study (Gustafsson et al., 2007; Côté, 1999). The absence of sex differences in profile membership is noteworthy in light of variable-centered reports of higher anxiety or burnout among girls in some samples; it suggests that psychological risk configurations may be similarly prevalent across genders in competitive settings that impose comparable demands. From an applied perspective, the training-hours finding suggests that monitoring load progression may be as informative as monitoring mood, particularly in early-specialization systems in which weekly volumes escalate quickly, and the absence of sex differences indicates that risk monitoring should be applied uniformly to boys and girls rather than targeted at one sex.

Practically, the findings point to the potential of profile-based risk monitoring, pending prospective validation of its predictive performance. Brief multicomponent monitoring—for example, periodic administration of short forms of the ABQ, an amotivation checklist, and the SAS-2—could eventually help identify athletes whose scores cluster in the high-risk configuration, and such flags could trigger supportive rather than disciplinary responses: adjusting training loads, restoring perceived competence and autonomy through mastery-focused climates, and addressing worry through basic psychological skills training (DeFreese and Smith, 2013; Ryan and Deci, 2017; Raedeke and Smith, 2004). These applications remain to be validated prospectively and should not be treated as a validated screening tool at this stage. Given that the moderate-risk profile already showed a dropout rate more than double that of the low-risk profile, future intervention research should not be reserved for the highest-risk athletes alone.

Several limitations qualify these conclusions. First, although dropout was recorded prospectively, the psychological indicators were measured at a single occasion, so changes in risk configurations across the season could not be modeled; latent transition analysis across multiple waves would be a valuable next step (Collins and Lanza, 2010). Second, dropout was defined as withdrawal from organized competitive sport in the primary sport without re-enrollment elsewhere; sport-general versus sport-specific dropout distinctions could not be fully adjudicated (Balish et al., 2014). Moreover, dropout was recorded from institutional enrollment data without information on reasons for withdrawal, so psychologically driven attrition could not be distinguished from attrition due to injury, academic redirection, deselection, or family relocation. Third, the sample was drawn from Jiangsu Province, China, and findings may not generalize to other competitive systems, age groups, or cultures; in addition, athletes were recruited from 21 sports schools and clubs, and athletes within the same institution share training environments, a source of clustering that was not modeled in the present analyses; standard errors may therefore be modestly underestimated, and multilevel extensions of person-centered models are a valuable direction for future research. Fourth, although R3STEP corrects for classification error, it assumes that the auxiliary model is correctly specified; alternative approaches such as the BCH procedure could be used to cross-validate the estimates. Finally, unmeasured variables such as injury, coach behavior, and family support likely contribute to both profile membership and dropout and merit inclusion in future multilevel models (Balish et al., 2014). In addition, sport type and competitive level were not included as covariates, competition load and basic psychological need satisfaction were not measured, external and introjected forms of regulation were not assessed, no study-specific confirmatory factor analysis of the measures was conducted, and measurement invariance across sex was not tested; age was modeled as a linear covariate, so potential non-linear age effects across the 12–17 year range also remain unexamined. Finally, the observational design cannot establish that profile membership caused dropout or identify the mechanisms through which withdrawal occurred.

In summary, this study shows that burnout, amotivation, and competitive anxiety cohere into identifiable risk profiles among adolescent athletes, that a minority of athletes occupy a convergent high-risk profile, and that dropout within a year is associated with a several-fold higher odds of membership in this profile. Person-centered risk assessment may provide a useful framework for future retention strategies, but its predictive performance and practical utility require further prospective validation.

## Data Availability

The raw data supporting the conclusions of this article will be made available by the authors, without undue reservation.
